# The Temporal Modulation of Nocebo Hyperalgesia in a Model of Sustained Pain

**DOI:** 10.3389/fpsyt.2022.807138

**Published:** 2022-03-23

**Authors:** Eleonora Maria Camerone, Simone Battista, Fabrizio Benedetti, Elisa Carlino, Lucia Grazia Sansone, Luca Buzzatti, Aldo Scafoglieri, Marco Testa

**Affiliations:** ^1^Department of Neurosciences, Rehabilitation, Ophthalmology, Genetics, Maternal and Child Health, University of Genova, Genoa, Italy; ^2^Department of Psychology, University of Milan-Bicocca, Milan, Italy; ^3^Department of Physiotherapy, Human Physiology and Anatomy, Experimental Anatomy Research Group, Vrije Universiteit Brussel (VUB), Brussels, Belgium; ^4^Department of Neuroscience, University of Turin Medical School, Turin, Italy; ^5^Plateau Rosà Laboratories, Plateau Rosà, Switzerland; ^6^School of Allied Health, Anglia Ruskin University (ARU), Cambridge, United Kingdom

**Keywords:** pain, nocebo hyperalgesia, expectation, temporal suggestions, sustained pain

## Abstract

**Background:**

The direction and the magnitude of verbal suggestions have been shown to be strong modulators of nocebo hyperalgesia, while little attention has been given to the role of their temporal content. Here, we investigate whether temporal suggestions modulate the timing of nocebo hyperalgesia in an experimental model of sustained pain.

**Methods:**

Fifty-one healthy participants were allocated to one of three groups. Participants received an inert cream and were instructed that the agent had either hyperalgesic properties setting in after 5 (Nocebo 5, N5) or 30 (Nocebo 30, N30) minutes from cream application, or hydrating properties (No Expectation Group, NE). Pain was induced by the Cold Pressure Test (CPT) which was repeated before cream application (baseline) and after 10 (Test10) and 35 (Test35) minutes. Changes in pain tolerance and in HR at each test point in respect to baseline were compared between the three groups.

**Results:**

Tolerance change at Test 10 (Δ10) was greater in N5 (MED = −36.8; IQR = 20.9) compared to NE (MED = −5.3; IQR = 22.4; *p* < 0.001) and N30 (MED = 0.0; IQR = 23.1; *p* < 0.001), showing that hyperalgesia was only present in the group that expected the effect of the cream to set in early. Tolerance change at Test 35 (Δ35) was greater in N5 (MED = −36.3; IQR = 35.3; *p* = 0.002) and in N30 (MED = −33.3; IQR = 34.8; *p* = 0.009) compared to NE, indicating delayed onset of hyperalgesia in N30, and sustained hyperalgesia in N5. No group differences were found for HR.

**Conclusions:**

Our study demonstrated that temporal expectations shift nocebo response onset in a model of sustained pain.

## Introduction

One's expectations of pain amelioration or worsening can significantly change pain perception, reducing and increasing its intensity, respectively (1). The impact of expectations on pain is evident in placebo analgesia and in nocebo hyperalgesia, where pain ameliorates or worsens following the administration of an inert treatment delivered in association with positive verbal suggestions for placebo (i.e., suggestions of pain decrease) and negative ones for nocebo (i.e., suggestions of pain rise) (2–4). Although placebo and nocebo effects can be induced in multiple ways—i.e., contextual factors including non-verbal communication, appearance of the medical personnel, clinical setting, type of intervention (5, 6)—and they can involve processes other than expectations—i.e., learning processes such as social observational learning, classical and operant conditioning (7, 8)—here we focus on verbal suggestions as the main factor inducing positive and negative expectations, which in turn are responsible for placebo and nocebo responses, respectively.

While the magnitude (9–11) and the direction (4, 12–14) of verbal suggestions have been identified as modulators of placebo analgesia and nocebo hyperalgesia, little attention has been given to the modulatory role of temporal suggestions, which was recently investigated for the first time by our team (15, 16).

In a recent experiment, we demonstrated, for the first time, that it is possible to “externally time” placebo and nocebo effects, meaning that their onset of action can be shifted in time by delivering different temporal suggestions. Precisely, we showed that by telling some participants that the administered (inert-) cream would set in after 5 min, the analgesic and hyperalgesic effects set in early, compared to the delayed effect reported by participants that were told that the (inert-)cream would set in after 15 and 30 min (15). In this previous study, pain was experimentally induced with short-lasting electrical stimuli of medium-to-low pain intensity (15). While this pain model has several advantages (e.g., safe, easy to induce, and consisting of short lasting pulses that can be repeated to collect more trials) and is therefore widely used in experimental pain research (17), this is not free from limitations. For instance, its clinical relevance has been questioned by some, arguing that clinical pain is rarely brief and precisely timed (18–21). Besides, this pivotal study relied on verbal pain reports, therefore the influence of report biases on self-reported pain ratings could not be excluded. In a subsequent experiment we demonstrated that the “external timing” of placebo analgesia persists in a model of sustained pain (16), while it is not known whether this temporal modulatory effect on sustained pain persists in the case of nocebo hyperalgesia.

In the present study, we investigated whether the finding that temporal suggestions modulate the onset of nocebo hyperalgesia on short-lasting, medium-to-low intensity electrical stimuli (15), extends to longer-lasting (tonic), higher-intensity pain induced with the Cold Pressor Test (CPT), a pain model which has been suggested to offer a good approximation of clinical pain (20, 21). Instead of solely relying on verbal pain ratings, as done in our previous work (15), we assessed maximum pain tolerance (i.e., operationalised as the time participants resisted with their hand in freezing-cold water) as behavioral outcome measure, avoiding the possible influence of report biases. While maximum pain tolerance is our primary outcome measure, we also measured pain ratings during the pain test, and we recorded participants' expectations toward the effectiveness of the cream retrospectively. In addition, since previous research has shown that heart rate (HR) increases during the pain anticipatory phase (22), we measured HR to detect nocebo-related anticipatory anxiety responses. At last, we also measured some psychological traits (i.e., personality, cognitive, and emotional factors) which have been previously linked to nocebo responsiveness [for overview see the recently published systematic review by Kern et al. (23)]. Compared to the placebo effect, less research has investigated psychological traits associated with nocebo responsiveness (23). However, traits such as high state and trait anxiety [assessed with state-trait anxiety inventory in Camerone et al. (15), Colloca et al. (24), and Corsi et al. (25)], fear of pain [assessed with the Fear of Pain Questionnaire in Aslaksen and Lyby (26)] and low optimism [assessed with the revised life oriented test in Geers et al. (27)] have been associated with greater nocebo responsiveness. In addition, high anxiety has been shown to be a predictor of enhanced pain perception [assessed with the Beck Anxiety Inventory in Kose-Ozlece et al. (28)]. Note that the Beck Anxiety Inventory can be described as a measure of prolonged state anxiety (29). Furthermore, the extent to which an individual is more inward or outward oriented seems to play a role in influencing placebo responsiveness [assessed with the behavioral inhibition/approach scales in Broelz et al. (30) and Darragh et al. (31)], while it is yet to be understood whether greater inward orientation is associated with enhanced nocebo responsiveness. In the present study, we used the same questionnaires of the forecited studies to clarify whether such personality traits influence nocebo responsiveness in an experimental model of sustained pain.

To sum up, the main aim of the present study is to investigate whether temporal information can modulate the onset of nocebo hyperalgesia in a model of sustained pain, induced with the CPT. Therefore, our primary outcome is the time taken by participants to reach the maximum pain tolerance during the pain test, while secondary outcomes include HR during the pain anticipatory phase and subjective pain ratings during the test. A secondary aim of the present study is to investigate whether retrospective participants' expectations of the cream efficacy and psychological factors are associated with nocebo responsiveness.

## Materials and Methods

### Participants

The study took place at the Experimental Anatomy Research Department at the Vrije Universiteit Brussel (VUB), Belgium. Sample size calculation has been calculated using G^*^Power (see Supplementary Material: Content 1). Forty-four healthy volunteers were recruited and randomized between the two experimental groups (i.e., nocebo groups), while participants of the control group (*N* = 17) were taken from our first experiment [(16); for further details see “Group allocation” section]. All participants were recruited both from the student population of the VUB (i.e., experimenter directly approached students around the university and asked them whether they were interested in taking part in the experiment) and from the general population (i.e., through different social media outlets such as Facebook). Participants were not compensated for their participation. Participants between 18 and 45 years of age were considered eligible to join the study. Participants that were in cure with antidepressants or anxiolytics, had a history of cardiovascular disease, and that suffered from psychiatric, neurological, chronic musculoskeletal, and pain-related disorders were not considered eligible to participate in the study. Moreover, we instructed the participants not to consume alcohol, caffeine-based drinks, supplements, and/or analgesic medications 12 h before the experiment. We informed participants that they would take part in a study investigating the time of action of a newly developed hyperalgesic cream. We disclosed the actual purpose of the study only after full data collection was completed (see Debriefing Section). Participants provided written informed consent agreeing to be debriefed with all the study details at the end of the experiment. All experimental procedures followed the policies and ethical principles of the Declaration of Helsinki. The Ethics Committee of the Vrije Universiteit Brussel approved this study (18/03/20; BUN1432020000002/I/U).

### Experimenters

The same experimenter was responsible for participants' enrolment and testing in the two nocebo groups. The experimenter was a PhD student (University of Genova) of 26 years old who identified himself as male. The experimenter that collected the data of the control group [i.e., placebo analgesia study; (16)], was a PhD student (University of Genova) of 26 years old who identified herself as female. The experimenters were properly trained to run the experiment and they were both part of the same research group. The experimenters, both in the nocebo groups and the control one, were fully aware of the nature of the experiment (i.e., they knew the purpose of the study, they knew that the cream was sham, and they were not blind to group allocation).

### Group Allocation

The present study is a two-arm randomized trial with an external control group (32). Participants were randomly assigned to two nocebo groups (allocation ratio 1:1) using computer-generated random numbers lists with simple randomisation (www.random.org). As for the control group (i.e., external control group), this was taken from our previous experiment (16) in which participants were also randomised to one of three groups (i.e., Placebo 5, Placebo 30, and Control). This experiment is one of two studies examining the temporal onset of placebo and nocebo effects. The first experiment investigated the placebo effect (16), while the second one, here reported, studied the nocebo phenomenon.

The recruitment and testing for the two nocebo groups took place between April and July, 2020, while for the control group this occurred between June and July 2019. For further details on the decision of using the same control group of our previous experiment, please see Supplementary Material: Content 2.

#### Nocebo Groups

Participants in the two nocebo groups were instructed that the cream had hyperalgesic properties that would increase the painful sensation induced during the CPT (i.e., in truth, the cream was an inert substance). We provided both groups with specific details about the onset of action of the hyperalgesic cream.

Participants allocated to the Nocebo 5 group (N5) were told that the hyperalgesic effect would arise after 5 min from cream application, mimicking a fast-acting drug. They received the following instructions: “*The agent you will receive is known to have a strong hyperalgesic effect which sets in after 5 min from its application. You will, therefore, become more sensitive to pain and be able to keep your hand in the cold water for a shorter time in the two test sessions after 10 and 35 min* [experimenter points at time 10 and 35 min marks on a clock] *compared to the first test* [CPT baseline].”

Participants allocated to the Nocebo 30 group (N30) were told that the hyperalgesic effect would set in 30 min from cream application. Specifically, the following instructions were given: “*The agent you will receive is known to have a strong hyperalgesic effect which sets in after 30 min from its application. You will, therefore, become more sensitive to pain and be able to keep your hand in the cold water for a shorter time in the test session after 35 min* [experimenter points at time 35 min marks on a clock] *compared to the first test* [points at CPT baseline] *and the second test after 10 min* [points at Test 10].”

Note that the CPT was performed 10 and 35 min after cream application and not after 5 and 30 min, which were the specific time points at which participants expected the cream to set in (at 5 min for N5 and at 30 min for N30). We allowed a 5-min leeway to avoid participants doubting that the effect of a cream could be so precisely timed (i.e., setting in exactly after 5 and 30 min).

#### Control Group

Participants that were assigned to the control group were informed that they would receive an inert cream (No Expectation, NE): “*The agent you will receive is an inert cream that only has hydrating properties but no effect on pain perception. Therefore, your test performance after 10 and 35 min* [experimenter points at time 10 and 35 min marks on a clock] *may be similar to the performance in the first test* [CPT baseline], *but it can also be longer or shorter than before.”*

### Experimental Protocol

**Figure 1:**
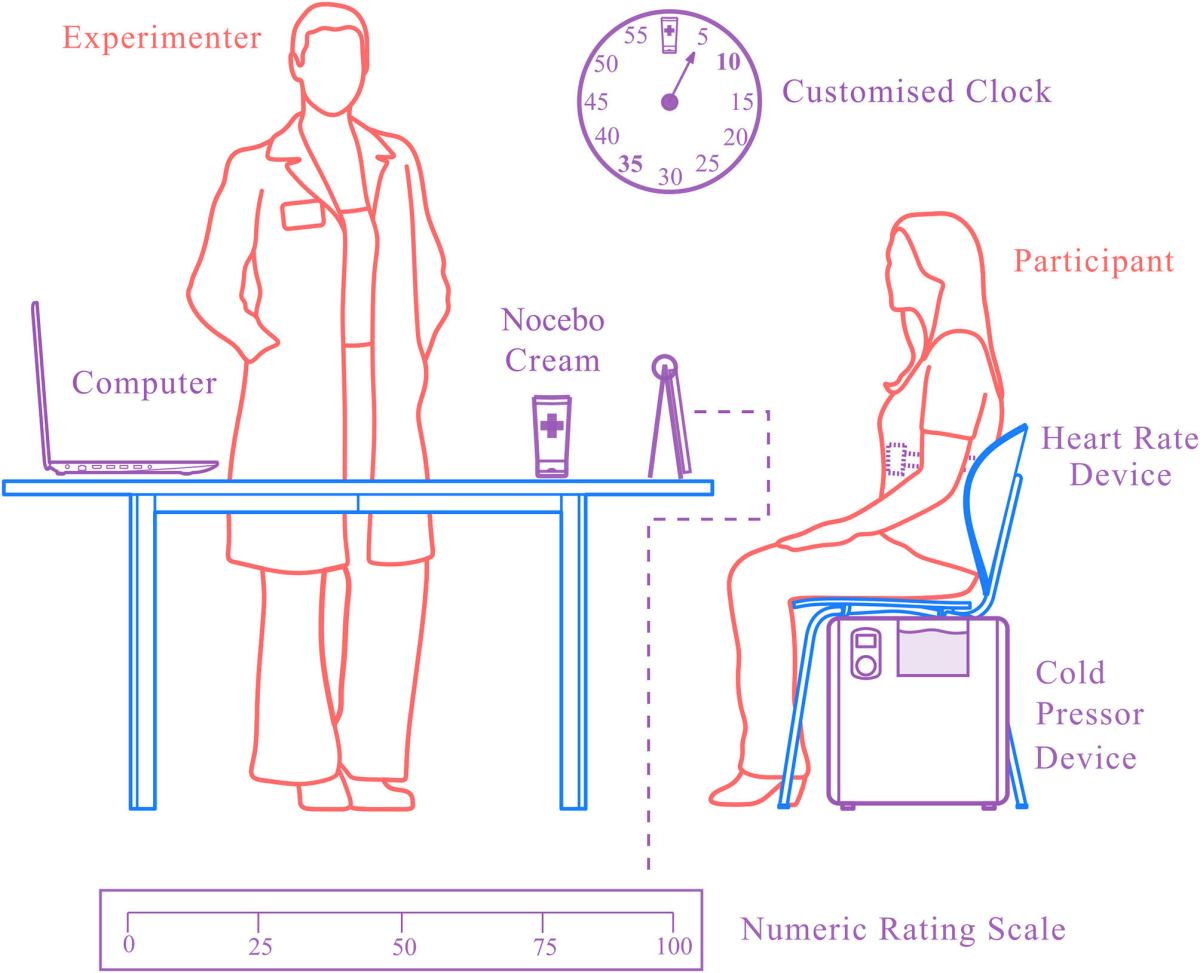
Experimental setting.

After providing written informed consent, participants were asked to sit on a chair positioned next to the CPT device. The investigator used a stopwatch displayed on a computer screen in front of the participants as well as a customized wall clock for participants' temporal orientation. The wall clock with 5-min intervals (i.e., 5–55) showed an icon of a cream tube at the 12 o'clock position to indicate the time-point of application of the cream (Figure 1).

**Figure 2:**
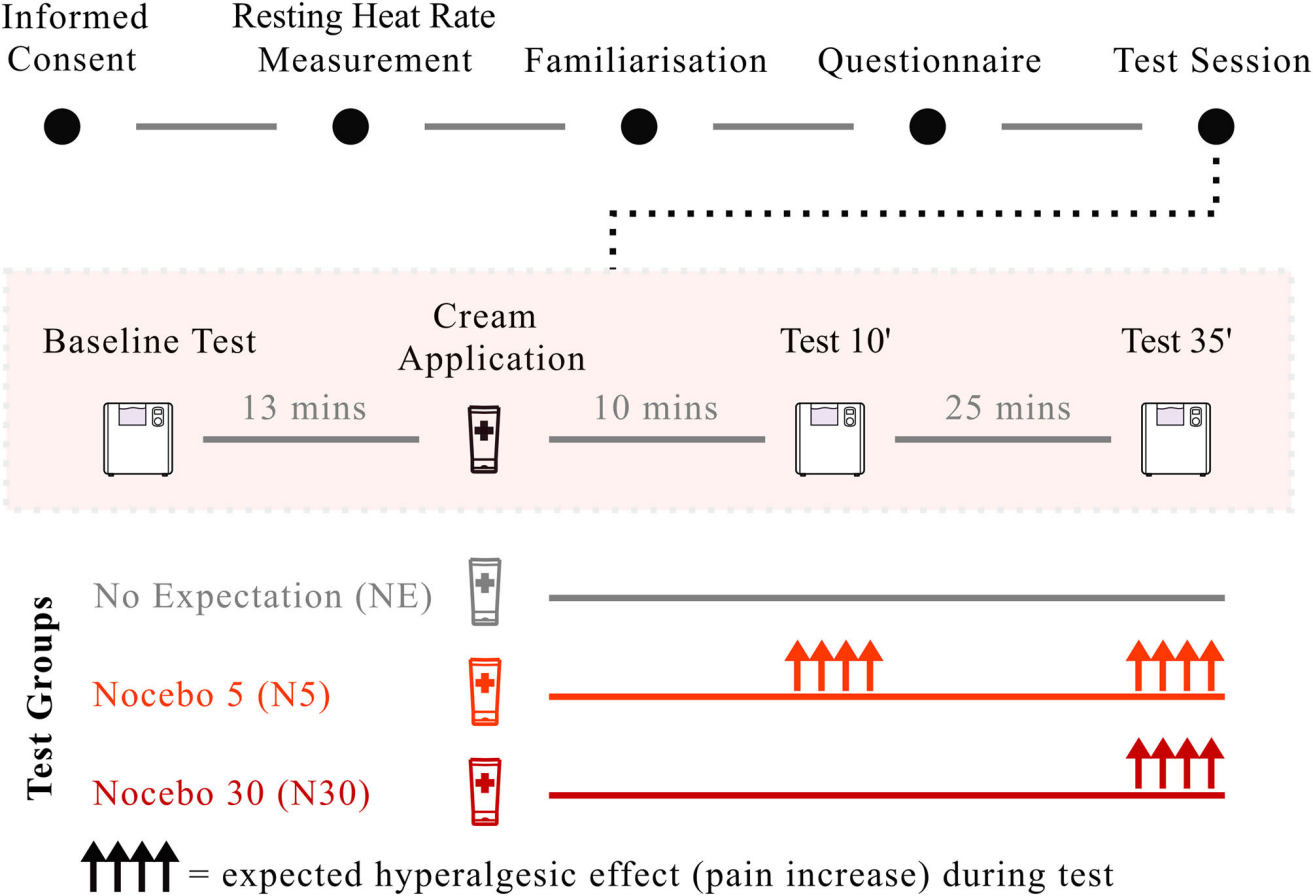
Study paradigm. After giving consent, participants' heart rate at rest was measured for 4 min. Participants completed the CPT familiarisation run and filled in the psychological questionnaires. After the CPT baseline test, the cream was applied along with suggestions of hyperalgesia (N5, Bright red; N30, Dark red) and neutral suggestions (NE), depending on group randomisation. Application of the cream and the delivery of suggestions took ~2 min. The CPT was then repeated after 10 and 35 min from cream application. Nocebo hyperalgesia, visualised as upper-facing arrows in the image, was expected both at Test 10 and at Test 35 for N5, and only at Test 35 for N30. No effect was expected for NE.

The experiment started with a 4-min heart rate measurement at rest, during which participants were asked to relax and breathe naturally. After instructing participants on how to perform the CPT task, they completed a familiarization trial. After the CPT familiarisation trial, all participants underwent the CPT baseline test, followed by participants' randomisation to groups and cream application. Along with cream administration, the experimenter provided participants with information about the nature of the cream (hyperalgesic cream in both nocebo groups and inert cream in the control group) and informed them about the expected onset of the hyperalgesic cream (nocebo groups only). Simultaneously with the application of the cream, the experimenter adjusted the customized wall-clock so that the minute hand pointed at the noon position, indicating the time of cream application (“Time 0”). CPT was then repeated 10 (Test 10) and 35 (Test 35) minutes from cream application (“Time 0”) (Figure 2). To be clear, the cream was not applied prior to each hand immersion, but it was only applied once, after the baseline CPT. Overall, the CPT was repeated a total of four times (familiarization, baseline, Test 10, Test 35) with a break of approximately 25 min between tests to restore the baseline hand temperature (Figure 2). During these breaks, participants filled in the psychological questionnaires (See Section: “Assessment of pain-related psychological traits”) and once completed, they were allowed to read or study, but were asked not to use their phones. The reason why participants were asked to complete the questionnaires during the breaks, rather that before or after the experiment, was to minimise the duration of the experiment and to engage participants in the same task during these pauses. The experimenter was present throughout the experiment, including during the breaks between the pain tests. However, to avoid biases the experimenter was not allowed to speak with participants. If the volunteers asked questions or wanted to chat, the experimenter was instructed to tell them that they were not allowed to talk with them during this time so that the interaction with each, and every participant remained unvaried, and that all questions would be answered at the end of the experiment (i.e., exception if the participant wanted to discontinue the experiment for any reason. In this case the experimenter was allowed to speak with the participant; this never occurred).

### Cold Pressor Test

During the CPT, participants were asked to immerse their left hand in seven liters of circulating cold water [7C°, ±0.2C°; CPT device: Thermo Scientific model Haake A 10B, Haake SC 100; Thermo Fisher Scientific, Waltham, MA; procedure adapted from Mitchell et al. (33)]. The experimenter drew a red line from the participant's ulnar to the radial styloid process (wrist level) to indicate the level to which participants had to lower their hand.

Before starting the CPT, 1 min of HR at rest was recorded. Ten seconds before the beginning of the test, participants were prompted by the experimenter to get ready (i.e., experimenter said, “Get ready!”) and to place their hand above the CPT device, showing readiness to immersion. Upon a verbal prompt from the experimenter (“Go”), the participant lowered their hand into the CPT device. The experimenter started the stopwatch to record the time between the beginning of exposure and hand withdrawal. The stopwatch was displayed on a computer screen located in front of the participant for temporal orientation. Participants were instructed not to move their fingers or hand while in the water and to keep their fingers spread with the palm parallel to the bottom of the device without touching it. For safety reasons, 10 min were set as the maximum time participants were allowed to spend with their hand in the water (34, 35), after which the test was discontinued, and the experiment ended. During CPT, subjective pain ratings were recorded every 15 s. The experimenter asked participants to quantify the pain they were experiencing on a scale from 0 (no pain) to 100 (unbearable pain) (see Section: Pain intensity ratings). Once pain became unbearable, participants removed their hand from the water basin and rested it on a towel placed on their knees. The time elapsed between hand immersion and withdrawal was recorded as CPT tolerance. The CPT, as described in this section, was repeated a total of four times during the experiment—i.e., familiarization, baseline, Test 10, Test 35—with no differences in the procedure between the familiarization trial and the other test sessions (i.e., baseline, Test 10, Test 35).

### Pain Intensity Ratings

To facilitate participants' self-reporting of pain during CPT, a poster depicting the rating scale was placed in front of them, which included verbal and numerical anchors (0 = not painful at all, 25 = somewhat painful, 50 = moderately painful, 75 = very painful, 100 = unbearable pain) (Figure 1). Despite verbal pain ratings were recorded every 15 s, the last pain score was taken at the moment of hand withdrawal to ensure that the maximum tolerance level was reached (i.e., this was the case for the two nocebo groups, but not for the control group, in which the last pain rating was recorded at the last 15 s interval prior hand withdrawal).

### Heart Rate Recording

The electrocardiogram (ECG) signal was measured using an HR monitor (Polar V800, Polar Electro Oy, Kempele, Finland), connected to two standard surface electrodes positioned on the participant's sternum with a band. Data were collected at a sampling rate of 700 Hz/s. HR was recorded for 4 min during a rest period in which participants were asked to sit comfortably and breathe normally. HR recording started 1 min before each CPT and continued through the test until 2 min after its completion. To limit the HR artifacts that might arise from hyperventilation related to pain-response, participants were instructed to maintain a regular and relaxed breath during each test session.

### Assessment of Pain-Related Psychological Traits and Retrospective Expectancy

During the breaks between CPT trials, participants were asked to complete multiple questionnaires that had previously been shown to link nocebo responsiveness with given personality traits (see Introduction):

- Beck Anxiety Inventory (BAI) to test the level of anxiety (36).
- Behavioral avoidance/inhibition scale (BIS/BAS) to test individuals' predisposition to inner or outward orientation (37).
- Fear of Pain Questionnaire (FPQ) to test fear of pain (38).
- Revised Life Oriented Test (R-LOT) to test the degree of optimism (39).

At the end of the experiment, participants were asked to rate retrospectively, on a scale from 0 (= not at all) to 7 (= very much), where 4 (= neutral), how much they had expected the cream to affect (i) their pain during the experiment (“*When the cream was applied on your hand, did you expect it to make you feel more pain during the water task?”*), and (ii) their ability to keep their hand in cold water (“*When the cream was applied to your hand, did you expect it to make you last less with your hand in the water?”*). Participants were also asked to rate the extent to which they had believed the given information regarding the onset of the hyperalgesic effect *(“When the cream was applied on your hand, how much did you agree with the following statement: The cream will start to become effective after 5 min* (N5)/*The cream will start to become effective after 30 min* (N30)”).

### Cream

All participants received an inert cream which was applied to their dorsal and volar left hand. The cream consisted of a water-based gel (KY-gel Johnson&Johnson) and was presented to participants in a transparent plastic tube. The cream was applied on the palmar and dorsal side of participants' hand up until the red line which was drawn by the experimenter, and it was massaged into the skin for ~1 min to ensure full absorption.

### Debriefing

Participants were debriefed through an email sent once full data collection was completed. Here, we explained the actual purpose of the study, and clarified why deception had been necessary. Participants were invited to contact the experimenter if they felt the need to discuss their participation in the study or any other concerns. They were also reminded that they could withdraw their data if they wished. However, none of the participants decided to do so.

### Statistical Analysis

First, one-way ANOVA was run to test for baseline differences between the three groups in demographic parameters, and psychological constructs were assessed via the questionnaires. Data for CPT tolerance at baseline, after 10 (Test 10) and 35 (Test 35) minutes did not follow a normal distribution (Shapiro-Wilk tests *p* < 0.05), therefore non-parametric tests were used. Second, Friedman Tests were performed to detect differences in tolerance time across CPT trials at the three different time points (Baseline, Test 10 and Test 35) within each group. Data are presented as median ± interquartile range and the significance level was set at *p* < 0.05. Significant results were followed up using Wilcoxon Signed-Rank Tests. Significance acceptance level for pairwise comparison was adjusted for the number of comparisons (k=3) using the Bonferroni Correction (α/k), resulting in *p* = 0.017. Third, Kruskal-Wallis H-Tests were used for the between-group analysis. To this end, percentage change in pain tolerance from baseline were calculated (Δ_10_, Δ_35_) to compare the groups on values that were more standardized than raw scores. Percentage change in pain tolerance from baseline to Test 10 (Δ_10_) and Test 35 (Δ_35_) was calculated as follow:

Δ_10_ = (Test 10^*^100)/Baseline -100;

Δ_35_ = (Test 35^*^100)/Baseline -100.

Data are presented as median ± interquartile range and the significance level was set at *p* < 0.05. Significant results were followed up using pairwise Mann-Whitney *U*-Tests. Significance acceptance level for pairwise comparison was adjusted for the number of comparisons (*k* = 3) using the Bonferroni Correction (α/k), resulting in *p* = 0.017. Effect sizes were calculated as *r* = z/√N (40). The effect size measures between the groups were used to assess the actual power of the study in percentage, based on the data of the trial. A threshold > 80% was set as satisfactory. Fourth, pain rating analysis was performed. We calculated the slope of pain ratings as a function of time; the steeper the slope, the faster maximum pain tolerance was reached. Since the first pain rating was recorded after 15 s from the beginning of the CPT, participants that lasted <15 s would only have one pain score (i.e., the one reported at the moment of hand withdrawal). Since the nocebo manipulation aimed at reducing the tolerance time, six participants (i.e., five in the nocebo groups and one in the NE group) ended up lasting <15 s in at least one of the test sessions, which means that they would only have one pain rating, making it impossible to calculate the slope (i.e., at least two scores are needed to calculate a slope). Not considering this data would be a bias because it would mean excluding those participants that reached maximum pain tolerance faster, possibly because of the nocebo intervention. To avoid losing meaningful data, we have added to all participants an extra datapoint at time 0 in which we assumed 0 pain, ensuring that everyone has at least one pain rating at the beginning of the test (i.e., time 0) and one pain rating at the end of the test (i.e., moment of hand withdrawal for the nocebo groups; last 15 s interval for the NE group); this allowed us to calculated a slope for all participants (i.e., except for one participant in the NE group who lasted <15 s and for whom we do not have the pain rating at the moment of hand withdrawal). Pain ratings slopes scores did not follow a normal distribution (Shapiro-Wilk tests *p* < 0.05), therefore non-parametric tests were used. Friedman Tests were performed to detect differences in the slope across CPT trials at the three different time points (Baseline, Test 10, and Test 35) within each group. Also in this case, data are presented as median ± interquartile range and the significance level was set at *p* < 0.05 and significant results were followed up using Wilcoxon Signed-Rank Tests. Significance acceptance level for pairwise comparison was adjusted for the number of comparisons (*k* = 3) using the Bonferroni Correction (α/k), resulting in *p* = 0.017.

Fifth, correlation analysis (i.e., Pearson correlation) was conducted to investigate the relationship between retrospective expectancy in nocebo groups and Δ10 and Δ35. Retrospective expectations included participants' expectations of (i) pain, (ii) tolerance, and (iii) cream onset of action. In addition, mean and SD for retrospective expectations measures were calculated to check whether participants' expectations were in line with the instructions given by the experimenter at the earlier stage (i.e., check that expectations were successfully induced).

Sixth, correlation analyses (i.e., Pearson correlation) were performed to explore the relationship between participants' psychological traits and nocebo effects. Specifically, correlations between psychological traits in nocebo groups and Δ10 and Δ35 were investigated.

Lastly, since heart rate data followed a normal distribution (Shapiro-Wilk tests *p* > 0.05), parametric analysis was performed. Mean HR was computed for the 10 s that preceded the beginning of the CPT, allowing us to assess HR during the anticipatory phase before the test session (Anticipatory HR). Anticipatory HR was calculated for each test, resulting in three mean indices for each participant (Anticipatory HR Baseline; Anticipatory HR Test 10; Anticipatory HR Test 35). A three-way mixed ANOVA was run, with the within factor TIME (Anticipatory HR Baseline; Anticipatory HR Test 10; Anticipatory HR Test 35) and the between factor GROUP (N5, N30, NE). In addition, for each test session, the mean HR value was calculated by averaging HR measurements over the first 10 s, resulting in three mean indices (HR Baseline; HR Test 10; HR Test 35). We selected the first 10 s because this was the shorter tolerance score across participants, allowing us to have a parameter for all participants. A three-way mixed ANOVA was run, with the within factor TIME (HR Baseline; HR Test 10; HR Test 35) and the between factor GROUP (N5, N30, NE). Significant results were followed up using Bonferroni-corrected *t*-tests.

## Results

**Figure 3:**
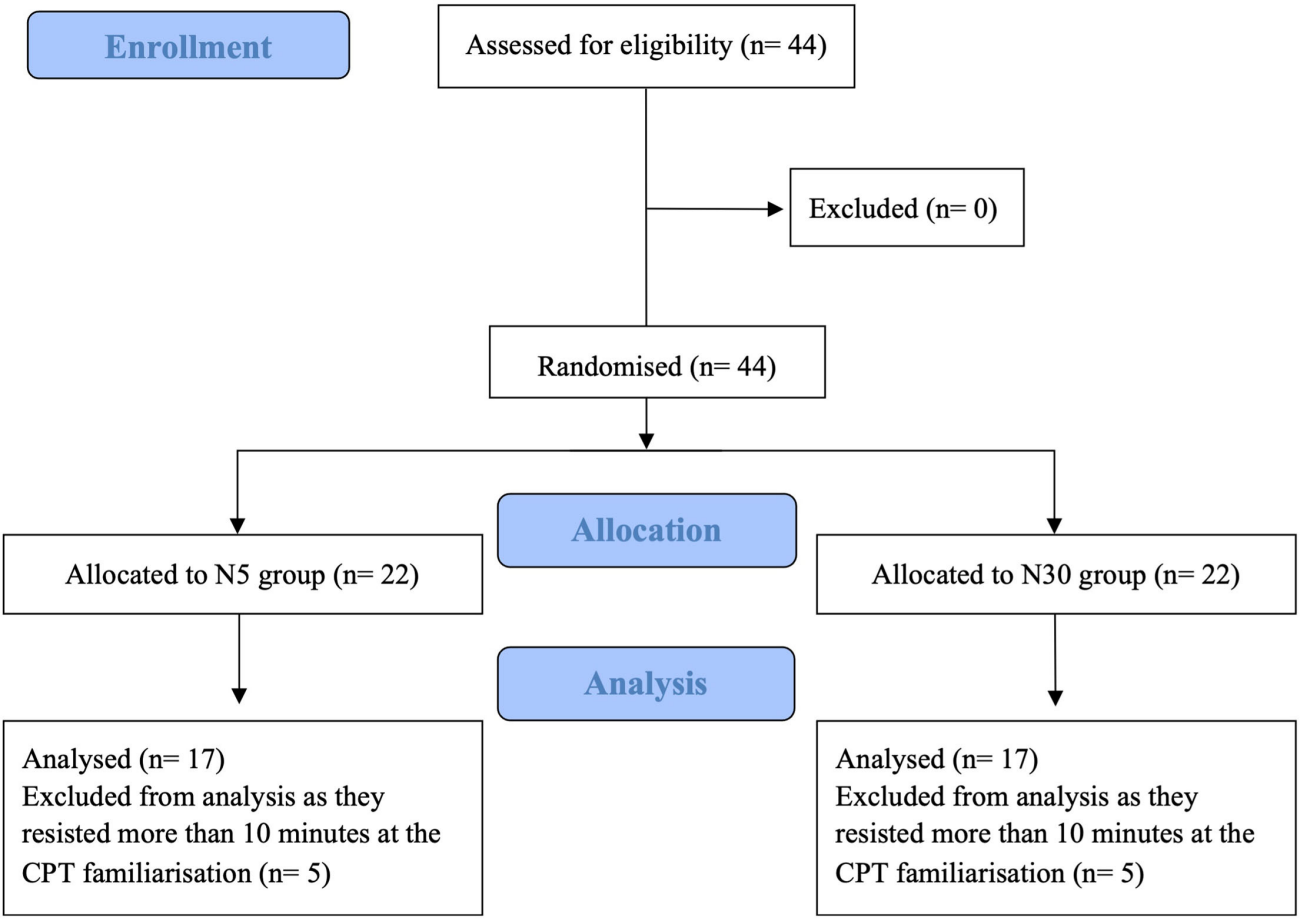
CONSORT flow-diagram.

We recruited 44 participants, 10 of which had to be excluded since they exceeded the maximum exposure time allowed with their hand into freezing-cold water (Figure 3). We relied on the same control group (*N* = 17) recruited beforehand for our study on placebo, resulting in a final sample size of 51 participants. One-way ANOVA and Chi-Square tests showed no baseline groups differences (*p* > 0.05) with respect to age, BMI, gender, and key psychological traits (Table 1). Kruskal-Wallis H-Test showed no significant baseline differences between groups in CPT tolerance (*p* = 0.237).

**Table 1:** Participants' descriptive characteristics and psychological traits.

**Groups**	**NE**	**N5**	**N30**
N	17	17	17
Age (Mean ± SD)	28.3 ± 3.4	24.3 ± 3.9	27.2 ± 4.6
BMI (Mean ± SD)	24.4 ± 2.5	24.1 ± 3.7	24.0 ± 2.3
Sex (F(%);M(%))	7 (41.2);10 (58.8)	9 (52.9);8 (47.1)	11 (64.7);6 (35.3)
Handedness (R(%))	13 (76.5)	17 (100.0)	17 (100.0)
BAI (Mean ± SD)	10.4 ± 4.9	14.8 ± 11.9	14.0 ± 9.2
BAS-Drive (Mean ± SD)	8.8 ± 2.3	8.8 ± 2.1	9.0 ± 1.7
BAS-Fun-Seeking (Mean ± SD)	8.1 ± 1.9	8.2 ± 2.1	8.8 ± 1.8
BAS-Reward (Mean ± SD)	8.3 ± 2.1	7.5 ± 2.1	8.0 ± 1.8
BIS (Mean ± SD)	14.6 ± 2.1	13.7 ± 3.3	13.2 ± 3.9
FPQ (Mean ± SD)	72.4 ± 12.9	71.3 ± 18.1	78.9 ± 14.2
RLoT (Mean ± SD)	14.3 ± 4.1	13.8 ± 5.6	15.1 ± 3.5

*SD, Standard Deviation; BMI, Body Mass Index; M, Male; F, Female; R, Right; BAI, Beck Anxiety Inventory; BAS, Behavioural Activation Scale; BIS, Behavioural Inhibition Scale; FPQ, Fear of Pain Questionnaire; RLoT, Life-Orientation Test-Revisited*.

### Nocebo Effects

Within-group analyses using Friedman Tests revealed, in both nocebo groups, a statistically significant difference in CPT tolerance depending on the temporal execution of the CPT test, either at baseline, after 10 (Test 10) or 35 (Test 35) minutes [Nocebo 5, $\chi_{\left(2\right)}^{2}$ = 15.394, *p* < 0.001; Nocebo 30, $\chi_{\left(2\right)}^{2}$ = 10.836, *p* =0.004] from cream application. Contrarily, no significant difference in CPT tolerance across time-points was shown in the NE group, $\chi_{\left(2\right)}^{2}$ = 2.471, *p* = 0.291. *Post-hoc* analyses were run using the Wilcoxon Signed Ranks tests (Table 2). N5 group showed a significant decrease in CPT tolerance at Test 10 (*p* = 0.001) and at Test 35 (*p* = 0.004) compared to baseline. No significant difference was shown in CPT tolerance between Test 10 and Test 35 (*p* > 0.05). N30 group showed no significant difference in CPT tolerance between Test 10 and baseline (*p* > 0.05). However, CPT tolerance significantly decreased at Test 35 compared to both baseline (*p* = 0.017) and Test 10 (*p* = 0.004).

**Table 2:** Median and interquartile range of CPT pain tolerance of all groups at the three test and within-group comparisons of CPT tolerance.

	**Baseline**		**Test 10**		**Test 35**	
	**Median**	**IQR**	**Median**	**IQR**	**Median**	**IQR**
NE	72.0	262.5	65.0	250.5	69.0	284.5
N5	57.0	112.5	38.0	91.5	50.0	85
N30	53.0	37	50.0	64	38.0	49.5
**Groups**	**Comparisons**		**Wilcoxon Signed rank test**		**Effect size**	**Power analysis**
NE	No *Post-hoc*		/		/	/
N5	T_10_ vs. Baseline		*Z* = −3.315, *p =* 0.001		*r =* 0.568	>80%
	T_35_ vs. Baseline		*Z* = −2.912, *p =* 0.004		*r =* 0.499	>80%
	T_10_ vs. T_35_		*Z* =-0.398, *p =* 0.691		*r =* 0.068	>80%
N30	T_10_ vs. Baseline		*Z* = −0.700, *p =* 0.484		*r =* 0.120	>80%
	T_35_ vs. Baseline		*Z* = −2.392, *p =* 0.017		*r =* 0.410	>80%
	T_10_ vs. T_35_		*Z* = 2.864, *p =* 0.004		*r =* 0.491	>80%

*IQR, Interquartile Range*.

**Figure 4:**
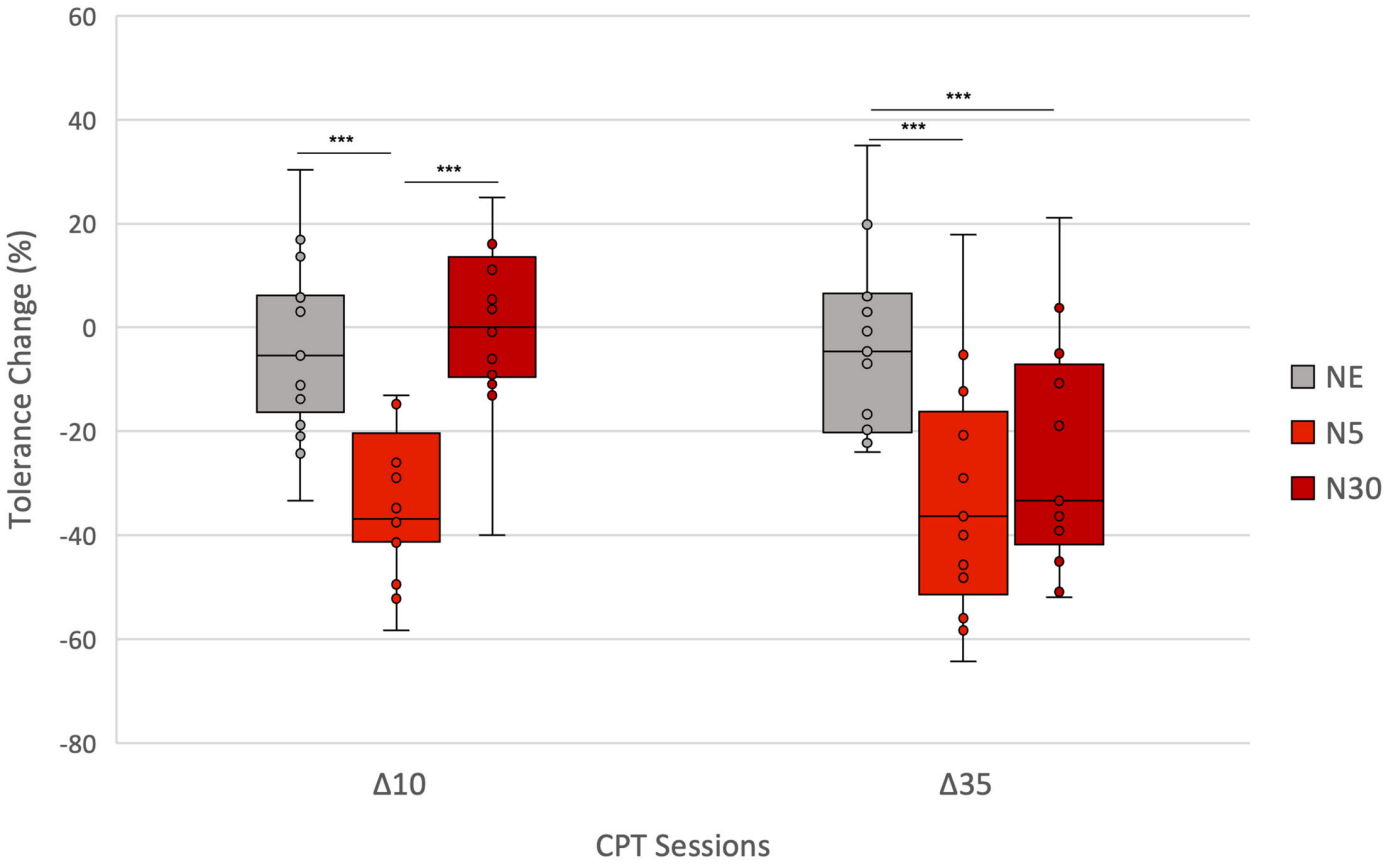
Between-group comparison: Percent change in CPT tolerance from Baseline to Test 10 (Δ10) and to Test 35 (Δ35) for each group (NE, N5, N30). Asterisks indicate significant differences in Δs between groups (*p < 0.05; **p < 0.01; ***p < 0.001). Δ10 was significantly lower in N5 than in both NE and N30. Δ35 was significantly lower in both N5 and N30 compared to the NE group. The lowest and highest boundaries of the boxes indicate the 25th and the 75th percentiles, respectively. The black line within each box indicates the median. Whiskers above and below the boxes indicate the largest and the lowest data points (excluding any outliers), respectively.

Between-group analysis using Kruskal-Wallis H-Tests showed a statistically significant difference in Δ_10_ between the different groups, $\chi_{\left(2\right)}^{2}$ = 18.1, *p* < 0.001. *Post-hoc* Mann-Whitney *U*-tests (Table 3) showed that Δ_10_ did not differ significantly between the NE group and N30 (*p* > 0.05). However, Δ_10_ was significantly higher in N5 than in both NE (*p* < 0.001) and N30 (*p* < 0.001). For Δ_35_, Kruskal-Wallis *H*-Test showed a statistically significant difference between groups, $\chi_{\left(2\right)}^{2}$ = 12.0, *p* = 0.002 (Table 3). *Post-hoc* Mann-Whitney *U*-tests (Table 3) revealed that Δ_35_ was significantly higher in both N5 (*p* < 0.002) and N30 (*p* < 0.009) compared to the NE group. No significant difference in Δ_35_ was found between N5 and N30 (*p* > 0.05) (Table 3). Figure 4 summarises between-group results employing box-plots representation.

**Table 3:** Median and interquartile range of percent change in CPT pain tolerance (Δ_10_,Δ_35_) in the three experimental groups and between-group comparisons of CPT percental tolerance change.

**Groups**		**Median**	**IQR**			**Median**	**IQR**
	**Δ_10_**				**Δ_35_**		
NE		−5.3	22.4			−4.6	26.8
N5		−36.8	20.9			−36.3	35.3
N30		0.0	23.1			−33.3	34.8
**Group comparisons**	**Dependent variable**	**Mann-Whitney** ***U*****-test**	**Effect size**	**Power analysis**
	**Δ_10_**			
NE vs. N5		*U* = 43.0, *p* < 0.001	*r =* 0.599	>80%
NE vs. N30		*U* = 107.0, *p =* 0.196	*r =* 0.221	>80%
N5 vs. N30		*U* = 38.0, *p* < 0.001	*r =* 0.629	>80%
	**Δ_35_**			
NE vs. N5		*U* = 53.0, *p =* 0.002	*r =* 0.541	>80%
NE vs N30		*U* = 69.0, *p =* 0.009	*r =* 0.446	>80%
N5 vs. N30		*U* = 112, *p =* 0.263	*r =* 0.192	>80%

*IQR, Interquartile Range*.

### NRS Ratings

**Figure 5:**
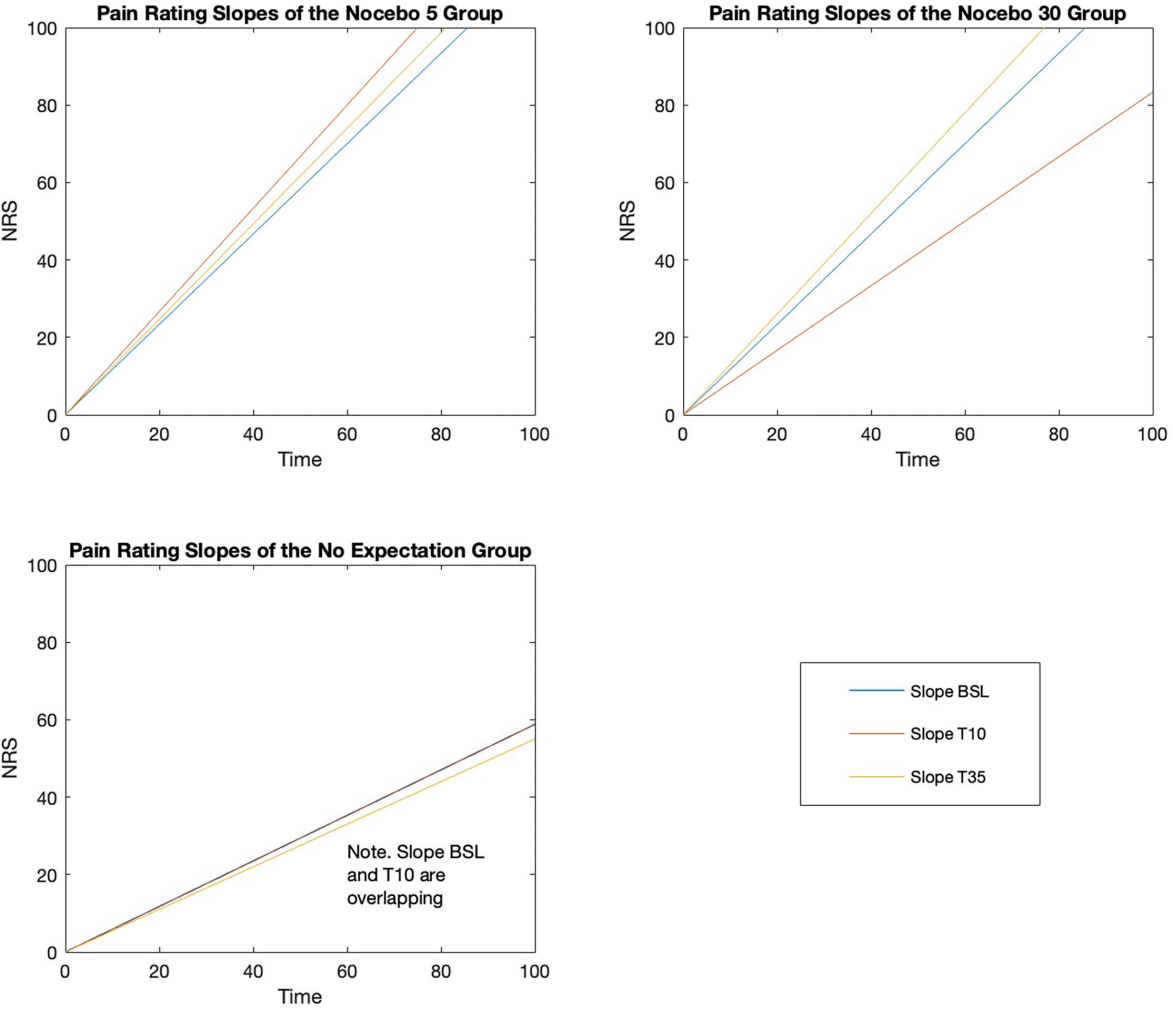
Pain rating slopes for the three groups at the three time-points (Baseline, Test 10, Test 35).

Within-group analyses using Friedman Tests showed, in both nocebo groups, a statistically significant difference in pain slope depending on when the CPT was performed, either at baseline, after 10 (Test 10) or 35 (Test 35) minutes from cream application [Nocebo 5, $\chi_{\left(2\right)}^{2}$ = 7.969, *p* = 0.019; Nocebo 30, $\chi_{\left(2\right)}^{2}$ = 10.062, *p* = 0.007]. Differently, the Friedman Test showed no significant difference in pain slope over time in the NE group [NE, $\chi_{\left(2\right)}^{2}$ = 0.561, *p* = 0.755]. *Post-hoc* analyses were run using the Wilcoxon Signed Ranks tests (Tables 4, 5) (Figure 5). N5 group showed a tendency (i.e., Bonferroni corrected *p* = 0.017) toward a significant increase in the steepness of the slope at Test 10 (*p* = 0.047) and at test 35 (*p* = 0.044) compared to baseline, while no significant difference in slope steepness was shown between Test 10 and Test 35 (*p* = 0.816). N30 group showed a tendency toward a significance decrease in slope steepness between baseline and Test 10 (*p* = 0.020). Importantly, an almost significant increase in slope steepness was shown when comparing the slope at Test 35 and at Baseline (*p* = 0.022) and a significant increase when comparing Test 35 with Test 10 (*p* = 0.008).

**Table 4:** Median and interquartile range of the slope of pain ratings at baseline, test 10 and Test 35 in the three experimental groups.

	**Slope baseline**		**Slope test 10**		**Slope test 35**	
	**Median**	**IQR**	**Median**	**IQR**	**Median**	**IQR**
NE	0.588	1.6	0.588	1.5	0.550	2.1
N5	1.167	1.1	1.333	1.1	1.233	1.1
N30	1.167	0.7	0.833	0.8	1.300	4.3

*IQR, Interquartile range*.

**Table 5:** Within-group comparison of the slope of pain ratings.

**Groups**	**Comparisons**	**Wilcoxon Signed rank test**	**Effect size**	**Power analysis**
NE	No *Post-hoc*	/	/	/
N5	T_10_ vs. Baseline	*Z* = −1.988, *p =* 0.047	*r =* 0.341	>80%
	T_35_ vs. Baseline	*Z* = −2.012, *p =* 0.044	*r =* 0.345	>80%
	T_10_ vs. T_35_	*Z* = −0.233, *p =* 0.816	*r =* 0.040	>80%
N30	T_10_ vs. Baseline	*Z* = −2.331, *p =* 0.020	*r =* 0.400	>80%
	T_35_ vs. Baseline	*Z* = −2.296, *p =* 0.022	*r =* 0.394	>80%
	T_10_ vs. T_35_	*Z* = −2.639, *p =* 0.008	*r =* 0.453	>80%

### Retrospective Expectancy and Psychological Tests

No significant correlations were shown, in either of the two nocebo groups, between retrospective expectations of (i) pain, (ii) tolerance, and (iii) cream onset of action and Δ_10_ and Δ_35_. However, considering that a rating of 4 indicates neutral expectations, the mean of retrospective expectations of (i) pain, (ii) tolerance, and (iii) cream onset of action indicates that participants had, on average, expectations somewhat in line (i.e., all average ratings > 4) with what they were told by the experimenter (Table 6).

**Table 6:** Participants' retrospective expectations.

**Groups**	**NE (Mean ±SD)**	**N5 (Mean ±SD)**	**N30 (Mean ±SD)**
Retro exp pain	n/a	4.8 ± 1.3	4.7 ± 1.7
Retro exp tolerance	n/a	5.1 ± 1.1	5.3 ± 1.6
Retro exp time	n/a	4.7 ± 1.9	4.4 ± 1.8
Average retro exp		4.9 ± 1.4	4.8 ± 1.7

No significant correlations were shown, in either one of the two nocebo groups, between the personality measures and Δ_10_ and Δ_35_.

### Heart Rate

Mixed-methods ANOVA showed no significant main effect of TIME, GROUP, nor of their interaction (*p* > 0.05) on anticipatory HR measures. Instead, a significant main effect of TIME on HR test measures (HR Baseline; HR Test 10; HR Test 35) was shown [*F*_(2, 96)_ = 6.601, *p* = 0.002], indicating that mean HR differed significantly across the three-time points (Baseline, Test 10, Test 35). Yet, no significant main effect of GROUP nor interaction between both factors were observed (both *p* > 0.05). *Post-hoc* pairwise comparison using the Bonferroni correction revealed that HR decreased significantly between baseline (*M* = 79.68, *SD* = 13.53) and Test 35 (*M* = 75.84, *SD* = 10.79) (*p* = 0.006), suggesting habituation to cold water. While still showing a tendency of HR decreasing over time, the other comparisons did not reach significance (*p* > 0.05).

## Discussion

Our previous study demonstrated that temporal suggestions modulate the onset of nocebo hyperalgesia on a phasic pain model, induced by short-lasting, medium-to-low intensity electrical pulses (15). Here, we extended these findings to a longer-lasting, higher-intensity, tonic pain model, and we relied on a behavioral outcome measure (i.e., maximum tolerance) instead of subjective pain ratings, as done in Camerone et al. (15). We replicated the main findings of our previous work, showing that the onset of nocebo hyperalgesia is dependent on the temporal suggestions that participants receive at the moment of (inert-)treatment administration [see Supplementary Material: Content 3 for the comparison of effect sizes of nocebo responses between the present study and Camerone et al. (15)]. Participants that were told that the cream had a fast time of action (N5) showed a decrease in tolerance level at the test session that took place soon after cream application (Test 10), demonstrating that suggestions of a fast-acting cream lead to early nocebo hyperalgesia onset. Differently, participants who were told that the cream would require a longer time before setting in (i.e., 30 min from application, N30) did not show a reduction in tolerance level at the early test session (Test 10), instead tolerance reduction set in at the delayed test trial (Test 35), showing that suggestions of delayed cream onset were responsible for postponing the hyperalgesic effect. This finding suggests that when giving a specific time tag to a predicted negative effect (in the present case, pain increase), this is likely to determine when such negative effect sets in. Although we did not directly measure trial-by-trial expectations, it is likely that the modulation of the onset of action of the nocebo cream was driven by participants' expectations, which were formulated accordingly with what they were told by the experimenter. In fact, the assessment of retrospective expectations indicated that participants had high expectations (~5 on a scale from 0 to 7, See Section on the Assessment of Retrospective Expectancy) that the cream would (i) increase their pain during the test, (ii) decrease their ability to last with the hand in the cold water, and (iii) set in at the time point suggested by the experimenter (after 5 min in N5 and after 30 min in N35). Given the modulatory role of expectancy on active treatments (13), it is likely that temporal verbal suggestions would have a similar modulatory effect on active treatments onsets, suggesting that maximum attention must be placed upon the temporal details that are given to patients when presenting them with a new intervention.

A second important finding of this study is that, once triggered, nocebo hyperalgesia remains stable over time (i.e., no difference was shown between Test 10 and Test 30 in the N5 group). This result is partially in line with our previous study which shows that once the nocebo response sets in, it increases over time (15). In both studies, the effect did not wear off over time. However, in one case (present study) it remained stable, while in the other it continued to increase (15). This discrepancy could be due to a “floor effect” which might have been present here (i.e., reduction of pain tolerance may reach a level after which lasting less time would mean barely keeping the hand in the water), but not in our previous study (i.e., NRS scores can keep increasing up until 10, even if no pain score ever got close). Alternatively, it could be due to the different methods of measuring pain, with a behavioral outcome in the first case, and with subjective ratings in the second. We suggest that the endurance of nocebo hyperalgesia over time is likely to be underpinned by the endurance of negative expectations (i.e., expectations that the hyperalgesic cream would reduce pain tolerance). Such argument is supported by the results of several nocebo studies which directly assessed trial-by-trial expectations and reported a correlation between expectations of high pain and enhanced pain perception (13, 41). Furthermore, Rodriguez-Raecke et al. have shown that negative expectations induced by verbal suggestions at day one, not only lead to pain worsening on that day, but also that this negative effect remains stable over the next 8 days (42). This study indicates that the endurance of nocebo hyperalgesia is associated with the endurance of negative expectations, indicating that, also in the present study, the endurance of nocebo hyperalgesia is likely to be attributed to the endurance of negative expectations. Accordingly, studies monitoring patients' recovery expectations from back pain onset during a 3-month (43) and a 2-week (44) period, have reported that expectations remained stable over time for most of the patients, and that the direction of expectations (i.e., positive, neutral, negative) was positively correlated with the therapeutic outcome. Altogether, our data is supported by previous research indicating that negative expectations are likely to endure over time (42–44). This underscores the importance of preventing the development of negative expectations in clinical routine when patients start new therapies, given that such expectations are likely to accompany the patient throughout the intervention, thus limiting, or in the worse cases abolishing, its positive effects (13, 43, 44).

Our findings are further supported by the pain ratings data. When the nocebo effect occurs, not only there is a decrease in pain tolerance, but maximum pain tolerance (assessed with pain ratings) is reached faster, as shown by a steeper pain ratings slope (see Statistical Analysis section for more details). Precisely, we found that in the N5 group, the pain rating slope was steeper at the time points in which the nocebo cream was told to be active (i.e., Test 10 and Test 35) compared to when not active (i.e., baseline), indicating that maximum pain tolerance was reached faster in the nocebo-modulated tests. Note that this difference in slope steepness between the nocebo tests and baseline was almost statistically significant. It is worth to highlight that we adjusted the comparison using Bonferroni correction which, if on one hand decreases the probability of “false positives” (i.e., type I error), on the other it increases the risk of not detecting real differences (i.e., type II error) (45). For what concerns the N30 group, a steeper pain rating slope was shown at the test occurring after 35 min—steeper slope at Test 30 compared to both baseline (i.e., almost significant) and Test 10 (i.e., significant), indicating that maximum pain tolerance was reached faster at the test in which the nocebo cream was expected to set in. Worth mentioning is that in this group, the slope was flatter at Test 10 compared to baseline (i.e., tendency to significance), indicating that when participants did not expect the nocebo cream to impair their tolerance, they were slower at reaching maximum pain. As opposed to the two nocebo groups, the pain rating slope remained stable over time in the NE group, indicating that maximum pain was reached with a similar speed when no nocebo suggestions where given. Although these results are promising, they are based on the within group analysis alone, and should therefore be taken with caution. On one hand, within group analysis allows to detect real differences that exist between the conditions which otherwise would stay undetected or covered by random noise (46). On the other hand, between group analysis is needed to draw conclusive remarks. In fact, the lack of the comparison with an external control group (as it would be in the between-group analysis) does not allow to rule out the possibility that the detected differences might be due to confounding factors (i.e., between-factor design allows for greater internal validity) (46). Unfortunately, between-group analysis for the pain ratings slopes was not possible because, due to differences in the nature of the data, slopes of the nocebo groups are not comparable with the slope of the NE. Indeed, the slopes of the nocebo groups are steeper because the last data point of the slope consisted in the maximum pain reached at the moment of hand withdrawal, which is when participants experienced the highest pain (all participants in the nocebo groups ended the pain test reporting NRS = 100). Differently, the slope of the NE group is flatter because the last data point of the slope consisted in the pain reached during the last 15-s interval prior to hand withdrawal, which is not when participants are experiencing the highest pain yet (on average participants reported NRS = 89).

For what concerns retrospective expectations, we found no significant correlations between these and our primary outcome (i.e., pain tolerance). However, measuring expectations retrospectively is an intrinsically biased measure because the reported expectations are reframed based on one's own experience. To have a more accurate representation of one's expectations, these should be assessed before each pain test (i.e., trial-by-trial assessment). However, this is challenging in placebo/nocebo research because repeatedly bringing attention to participants' expectation is likely to give out the true aim of the study (i.e., participants might question the real nature of the treatment), which is why we decided to assess expectations at the end of the study. The lack of a correlation between retrospective expectations and the primary outcome is in line with the results of our previous studies, also investigating the temporal component in nocebo hyperalgesia and placebo analgesia (15, 16). However, although the assessment of retrospective expectations did not lead to significant correlations, it allowed us to successfully check that participants developed expectations in line with what they were told by the experimenter—i.e., the average score of retrospective expectations was ~5 over 7 on a scale from 0 (= not at all) to 7 (= very much).

Regarding the psychological factors, no correlation was found between these, and our primary outcome measure. These findings are not particularly surprising given that the literature investigating which psychological factors can best predict nocebo responsiveness is rather scarce and discordant (23). In such an heterogeneous scenario, optimism/pessimism and fear/anxiety are, perhaps, the psychological factors that have been most often associated with an enhanced nocebo response (23). However, similarly to other recently published research (47, 48), we did not find a correlation between optimism/pessimism and nocebo responsiveness. For what concerns anxiety, most of the studies reporting a correlation, assessed anxiety with the state-trait anxiety inventory [e.g., Camerone et al. (15); Corsi et al. (25) found a correlation with trait anxiety; Colloca et al. (24) showed a correlation with both state and trait anxiety], while in the present study, we measured anxiety with the BAI, as done in the study of Kose-Ozlece et al. (28), in which a correlation between high anxiety and enhanced pain perception was reported. Therefore, the lack of correlation could be due to the assessment of anxiety with the BAI rather than with the state-trait anxiety inventory. It is worth pointing out that correlational analyses require much larger sample sizes than the one of this study [i.e., as suggested by Schönbrodt and Perugini (49) a typical scenario requires *n* = 250 for stable estimates], thus our results do not mean that correlations between the suggested psychological factors and nocebo responsiveness are not present, but that a larger sample size might be required to detect the effect. For instance, the study showing a correlation between anxiety measured with BAI and enhanced pain perception, included 140 participants (28). Yet, the primary aim of this study was the investigation of the temporal component of the nocebo effect, which is why reaching the appropriate sample size for correlational analyses was not a priority.

Considering heart rate data, no differences in HR were shown between groups, suggesting that nocebo hyperalgesia is not associated with HR changes. However, in line with our previous data, HR during the pain test decreased over time in all three groups, suggesting a physiological habituation response to the CPT (15). Lack of HR sensitivity as a physiological correlate of nocebo effects is in line with Daniali and Flaten (50) qualitative systematic review, in which heart rate variability, but not HR, was demonstrated to be a good physiological correlate of nocebo hyperalgesia (50). Also, anticipatory HR (i.e. HR during the 10 s that preceded hand immersion) did not differ between groups, and it remained stable over time, failing to pick up on anticipatory anxiety responses that are associated with nocebo hyperalgesia onsets (51). Our results contrast with Colloca and Benedetti (22)'s data that reported HR acceleration during the anticipatory phase before nocebo-cued noxious stimulations. Yet, the different type of noxious stimuli [electrical pulses in Colloca and Benedetti (22)] could account for the diverse anticipatory anxiety reactions, as well as for the associated HR responses.

Overall, the replication of our previous findings (15) on a model of tonic pain using the CPT is a step forward toward the understanding of the temporal modulation of clinical pain. It has in fact been argued that experimental pain induced with mild and short-lasting electrical pulses has limited resemblance with clinical pain, both in terms of stimuli duration and their level of aversiveness (19, 20). This is particularly true for non-continuous electrical stimulation [as in the case of single pulses repeated in time as done in Camerone et al. (15)], while greater clinical relevance is recognised to continuous electrical stimulation (17), indicating that stimulus duration is an important feature to mimic clinical pain. Oppositely, the CPT, despite still being far from clinical pain, has a longer duration and reaches higher intensity (i.e., maximum tolerance), leading to a sensation that is a better proxy to real-life pain (20, 21). In addition, given the ongoing “replication crisis” affecting natural sciences (52, 53), the successful replication of our previous results on a different type of experimentally induced pain adds value to the current study.

### Limitations

The empirical results reported herein should be considered in the light of some limitations. The first is the lack of a full randomisation of participants across the three groups. Instead, participants were randomised between the two experimental groups (N5 and N30), while the control group was collected at a different time point, as part of our previous experiment (16). This challenges the validity of the results of the between groups analysis for at least two reasons. First because the same pool of data (i.e., control group), has been analysed twice, increasing the risk of Type I error. However, to amend for this pitfall we have corrected for multiple comparison using a particularly conservative method, the Bonferroni correction (54), which is indicated as the test to use in those cases in which avoiding Type I error is imperative, as the present case (45). The second issue is that the experimenters differed between the two nocebo groups and the control group (See Section “Experimenters” in the Methods), adding a potential bias. For instance, the experimenter testing the control group was a female, while the experimenter of the two nocebo groups was a male, yet the experimenters were matched in terms of status—i.e., both with the same age (26 years old) and the same education (both PhD students). Since Kállai et al. (55) showed that greater experimenter status increases tolerance time, it is an advantage that our experimenters were matched in terms of theirs status (55). However, Kállai et al. (55) showed that healthy volunteers tolerate pain longer when they are tested by an experimenter of the opposite sex. This indicates that the differences in the gender of the experimenters in the present study might be a threat to the validity of the results. However, no significant baseline differences (*p* > 0.05) were reported in terms of tolerance time between the nocebo groups and the control one, demonstrating that such bias is not likely to be present in this study. The third issue is the time gap of almost 1 year between when participants in the control group (June and July, 2019) and those in the experimental groups (April to July, 2020) were tested. This is particularly concerning if we consider that the control group was collected before the start of the COVID-19 pandemic, while the nocebo groups were collected after its beginning. Hence, it is not possible to exclude that confounding factors related to this abnormal historical time, including the psychological and social challenges that people faced over this period, could have biased the study results. However, participants across the three groups were comparable (no baseline groups differences, *p* > 0.05) in terms of demographics (i.e., age, BMI, and gender) and psychological traits, including trait anxiety, optimism, fear of pain and individuals' motivational systems. Furthermore, it is important to highlight that if in the between-group analysis we compare each experimental group with the control one, this is not the case for the within-group analysis, in which the control condition is the baseline session within each group, considered independently. It follows that the issues related to the control group not being randomised, do not affect the results of the within group analysis. Such consistency between the results of the within and the between analyses, both suggesting that temporal suggestions modulate the onset of action of nocebo hyperalgesia, further supports the validity of the results of the between groups comparison despite the limitations associated with the non-randomised control group.

The second limitation concerns the lack of expectancy recording throughout the experiment, while participants' expectations were only measured retrospectively. On one hand, measuring expectancy retrospectively prevented participants' from questioning the true nature of the study. On the other hand, the lack of trial-by-trial expectations recording prevents our data from giving us information on the variation of temporal expectations over the course of the experiment. Since expectations update accordingly with (sensory) experiences, further research is needed to investigate the interplay between expectations updating and nocebo hyperalgesia temporal modulation.

### Recommendations for Future Studies

Future studies investigating the temporal modulation of nocebo hyperalgesia should first, measure trial-by-trial expectations to directly assess whether there is a direct association between one's hyperalgesic expectations at a specific time-point and the presence of the hyperalgesic effect at such time-point. Second, further research must investigate whether the shifts in time of nocebo hyperalgesia are associated with a neurophysiological response. As demonstrated by the present study, HR is not a good measure to detect nocebo hyperalgesia; future studies could consider using central measures such as electroencephalography and functional magnetic resonance imaging, which are effective at picking up signals associated with nocebo hyperalgesia (56, 57). Third, future designs should investigate whether the same temporal effects would be found with longer time-windows. While here we investigated a 35-min interval, it is not known whether temporal suggestions would have the same effect if the interval was of days, weeks, or months. At last, the modulation of temporal suggestions should be investigated on patients suffering from endogenous pain. In this context, the effect of temporal suggestions could be investigated directly on active treatments, by delivering different temporal suggestions regarding the expected onset of action of possible treatment side effects (i.e., informing the patient of the real side effects as it would normally be done, but giving different temporal indications).

## Conclusions

To conclude, we demonstrated that temporal suggestions modulate the onset of nocebo hyperalgesia, extending our previous findings to a model of tonic pain, relying on maximum pain tolerance as a behavioral outcome measure. Sometimes pain cannot be avoided but has to be tolerated, as in some cases of chronic pain (58–60). Therefore, understanding how to modulate one's tolerance levels can be particularly relevant in the clinical context (61). These results are promising, and further studies must build upon this evidence to better understand the influence of temporal expectations in the clinical setting and across diverse therapeutic interventions.

## Data Availability Statement

The raw data supporting the conclusions of this article will be made available by the authors, without undue reservation.

## Ethics Statement

The studies involving human participants were reviewed and approved by the Ethics Committee of the Vrije Universiteit Brussel. The participants provided their written informed consent to participate in this study.

## Author Contributions

EMC, FB, and EC conceived the presented idea. EMC collected part of the data, planned and performed the data analysis, and took the lead in writing the manuscript. SB collected and analysed the data and gave a significant contribution to the manuscript write up. AS supervised the practical development of the experiment assessing its feasibility from start to end. LS and LB was involved in data analysis and collection. MT supervised the project throughout, from the design of the experiment to the completion of the final manuscript. All authors discussed the results and contributed to the final version of the manuscript.

## Conflict of Interest

The authors declare that the research was conducted in the absence of any commercial or financial relationships that could be construed as a potential conflict of interest.

## Publisher's Note

All claims expressed in this article are solely those of the authors and do not necessarily represent those of their affiliated organizations, or those of the publisher, the editors and the reviewers. Any product that may be evaluated in this article, or claim that may be made by its manufacturer, is not guaranteed or endorsed by the publisher.
