# Supplementary material for: The Temporal Modulation of Nocebo Hyperalgesia in a Model of Sustained Pain
Source: Front Psychiatry. 2022 Mar 23;13:807138. doi: 10.3389/fpsyt.2022.807138 (PMC8983965; doi:10.3389/fpsyt.2022.807138)
Supplement: Supplementary file 1 [file Data_Sheet_1.pdf]

Supplementary Material

Content 1: Sample size calculation

A priori analysis was run with G*Power 3.1 to calculate the sample size needed. Based on ANOVA for repeated measure test, a sample of 42 participants was determined to accept a power of 80%, a significant level of 0.05 and an effect size of 0.41 (Petersen et al., 2014). By assuming a dropout rate of 20%, 50 participants were required to run the study.

Content 2: Control group collected separately from the experimental groups

The decision to rely on the previously collected control group was motivated by the difficulties we encountered due to the COVID-19 pandemic, which significantly delayed data collection. Such delay was particularly challenging since data was collected by an Italian researcher, Simone Battista, during his five months abroad as visiting PhD student at the Vrije Universiteit Brussel (VUB), and therefore the time-window to collect the data was limited. Data was promptly collected as soon as the pandemic restrictions were lifted, from April 2020. However, the limited remaining time to collect the data (until July 2020), and the flare-up of the pandemic made the recruitment particularly difficult since VUB students and staff, our primary source of recruitment, were working remotely and were, therefore, less prone to come to the University. Given the difficulties in recruitment and the time pressure to end the study, we decided to optimise data collection by relying on the control group collected during our previous experiment, since the two studies followed the same protocol for this group. Although the experimenter running the study was not the same for the nocebo groups and the control group, both experimenters followed the same experimental procedure and the same script, ensuring that the same instructions were given in both studies.

References:

Petersen, G. L., Finnerup, N. B., Colloca, L., Amanzio, M., Price, D. D., Jensen, T. S., & Vase, L. (2014). The magnitude of nocebo effects in pain: a meta-analysis. *Pain®*, *155*(8), 1426-1434.
